# Supplementary figures and images for: Targeted Mutational Analysis of Circulating Tumor DNA to Decipher Temporal Heterogeneity of High-Grade Serous Ovarian Cancer
Source: Cancers (Basel). 2022 Jul 29;14(15):3697. doi: 10.3390/cancers14153697 (PMC9367609; doi:10.3390/cancers14153697)

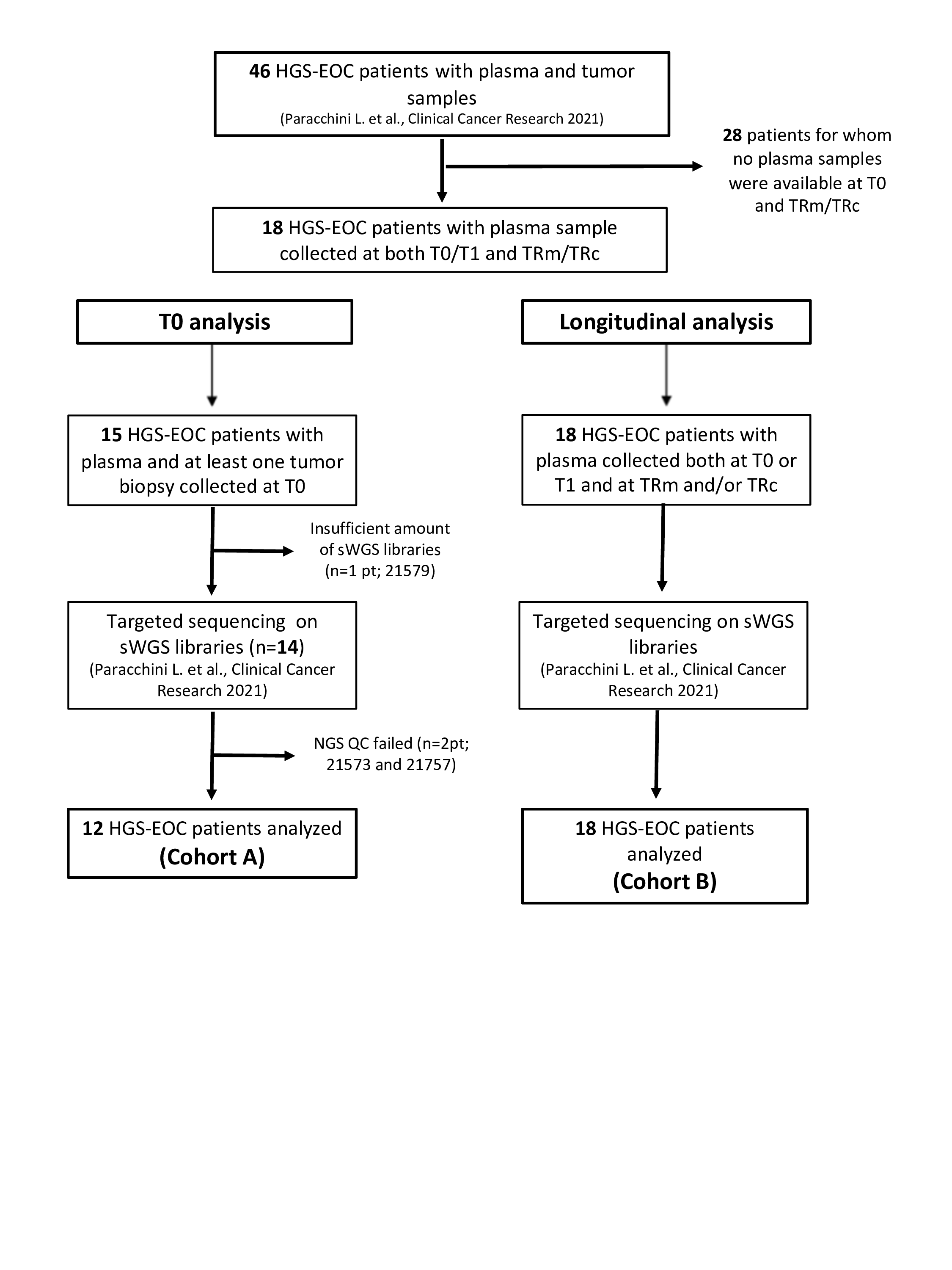

Supplement: Supplementary file 1 [file cancers-14-03697-s001.zip › cancers-1796307-supplementary/Supplementary/Supplementary_Figure_S1.png]

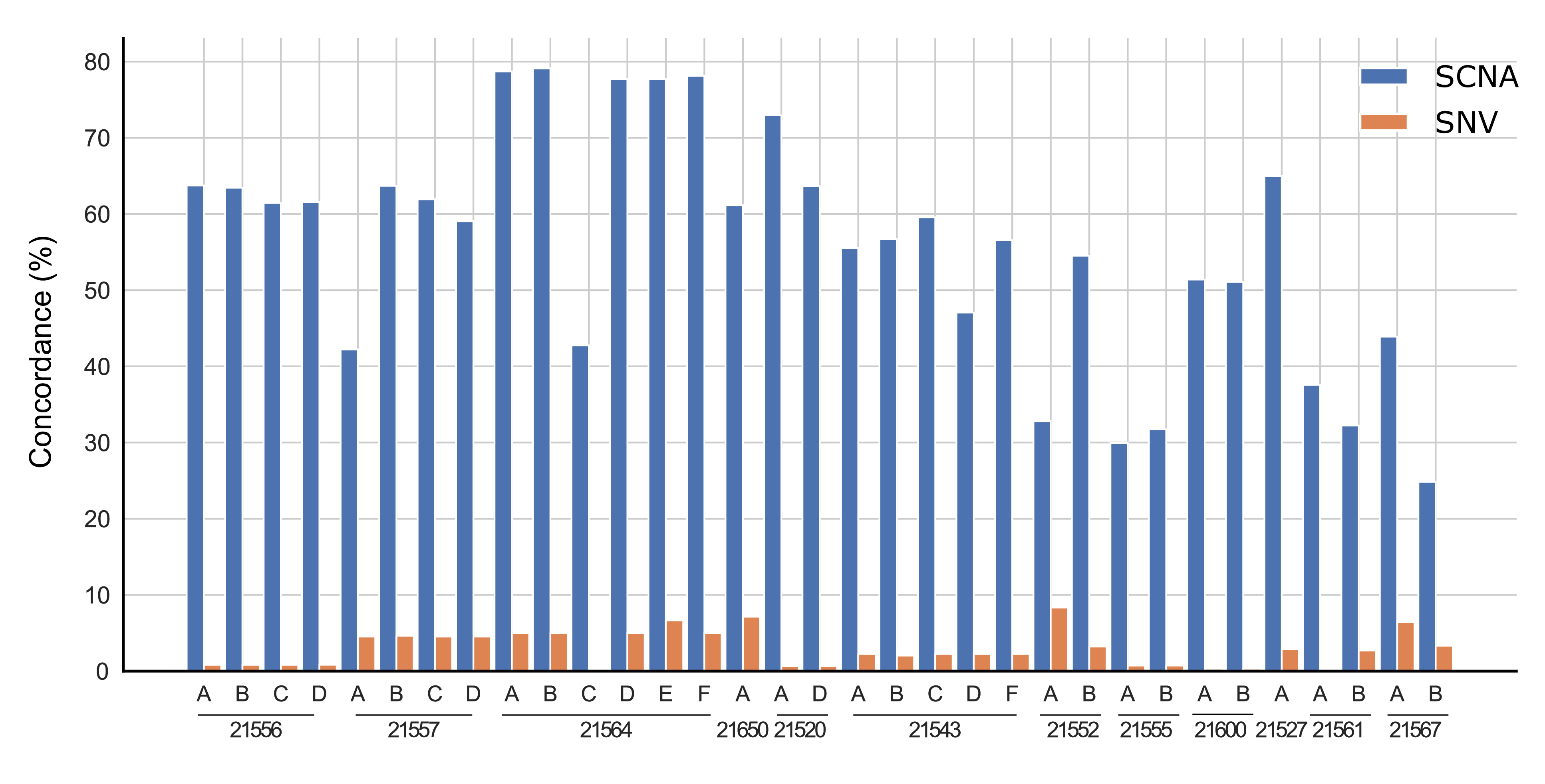

Supplement: Supplementary file 1 [file cancers-14-03697-s001.zip › cancers-1796307-supplementary/Supplementary/Supplementary_Figure_S2.png]

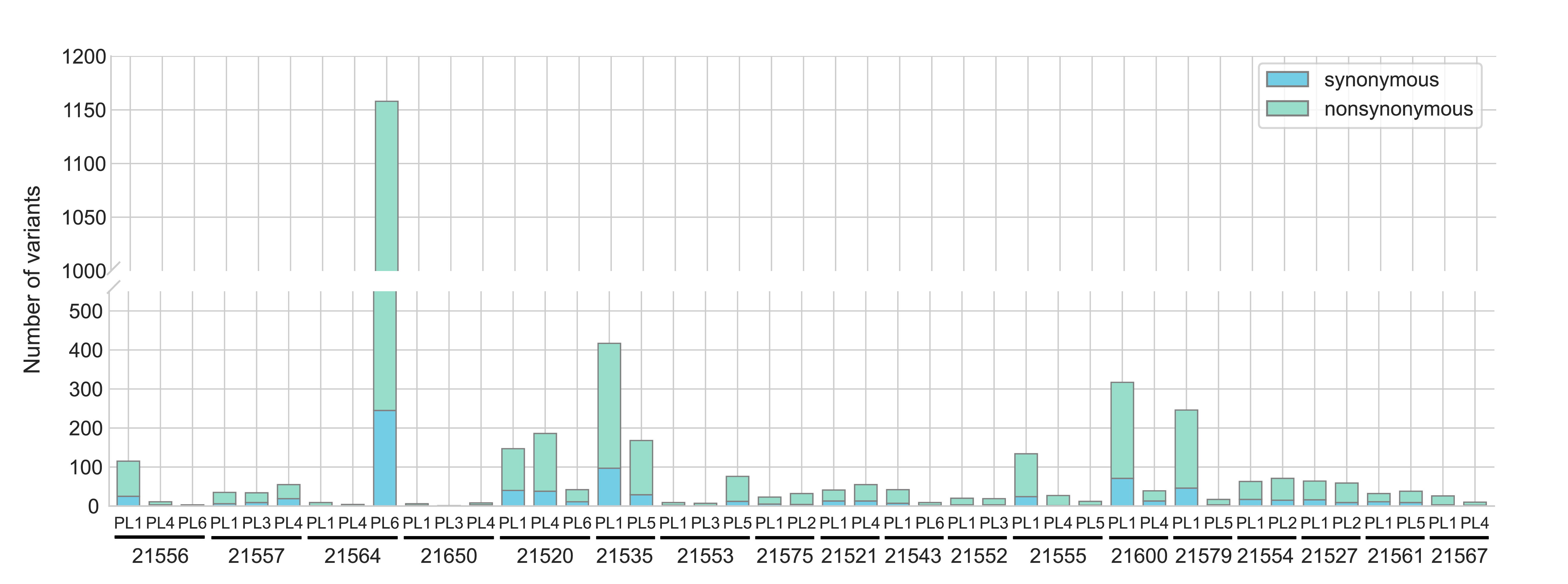

Supplement: Supplementary file 1 [file cancers-14-03697-s001.zip › cancers-1796307-supplementary/Supplementary/Supplementary_Figure_S3.png]

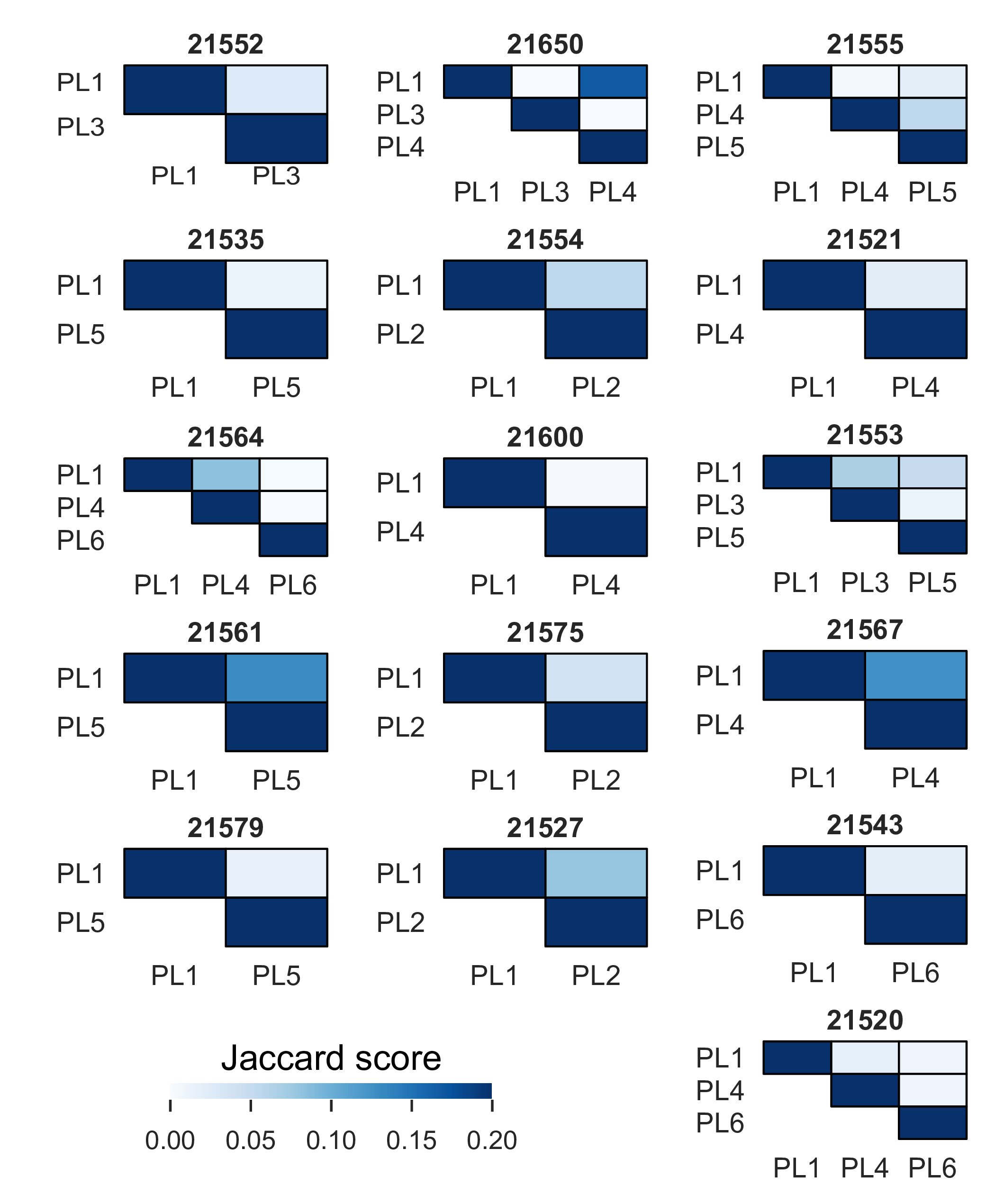

Supplement: Supplementary file 1 [file cancers-14-03697-s001.zip › cancers-1796307-supplementary/Supplementary/Supplementary_Figure_S4.png]
